# Physical activity: a neglected therapy for dementia

**DOI:** 10.1590/0102-311XEN216123

**Published:** 2024-10-18

**Authors:** Natan Feter, Jayne Feter, Gustavo S. Silva, Maria Inês Schmidt, Airton José Rombaldi

**Affiliations:** 1 Escola Superior de Educação Física, Universidade Federal de Pelotas, Pelotas, Brasil.; 2 Faculdade de Medicina, Universidade Federal do Rio Grande do Sul, Porto Alegre, Brasil.

## Introduction

Dementia is a leading cause of dependence and disability worldwide ^1^ ^,^ ^2^ ^,^ ^3^. The number of dementia cases worldwide is projected to increase from 57.4 million in 2019 to 152.8 million by 2050, with a new case of dementia being diagnosed every three seconds ^4^ ^,^ ^5^. In the last four decades, a great and continuous effort has led to the discovery of different pharmacological treatments to attenuate the symptoms and slow disease progression. For example, aducanumab and lecanemab are monoclonal antibodies directed against aggregated amyloid beta approved by the FDA (U.S. Food and Drug Administration), with findings indicating a reduction in amyloid-beta accumulation and slower cognitive decline through follow-up ^6^ ^,^ ^7^. However, these treatments are largely unavailable at the population level due to their high estimated annual cost ^8^.

A robust body of literature has established an association between physical activity and reduced risk of cognitive decline and dementia ^9^ ^,^ ^10^ ^,^ ^11^ ^,^ ^12^ ^,^ ^13^ ^,^ ^14^ ^,^ ^15^. Consequently, governmental and nongovernmental organizations have advocated the role of physical activity in mitigating the risk of dementia ^16^. For example, the World Health Organization’s (WHO) global action plan on dementia ^2^ recommends a minimum of 150 minutes of physical activity per week to prevent dementia. Similarly, national dementia plans, which are designed to reduce the current and projected burden of dementia in each country, endorse the role of physical activity as an essential preventive strategy. However, few studies have addressed the extent to which physical activity is recommended as a complementary treatment for dementia.

## Exercise as a prescription for people with dementia

Physical activity is a safe and effective nonpharmacological strategy to preserve cognition and functionality in people with intact cognitive function, mild cognitive impairment, and dementia ^9^ ^,^ ^17^ ^,^ ^18^. In a rapid search on PubMed combining dementia, physical activity or exercise, and meta-analysis keywords, a total of 278 records were identified in November 2023. By searching only meta-analysis with randomized controlled trials (RCTs) employing physical activity protocols in people with dementia, 12 studies were found ^19^ ^,^ ^20^ ^,^ ^21^ ^,^ ^22^ ^,^ ^23^ ^,^ ^24^ ^,^ ^25^ ^,^ ^26^ ^,^ ^27^ ^,^ ^28^ ^,^ ^29^ ^,^ ^30^. Also, three umbrella reviews of meta-analyses revealed that exercise could improve cognitive function in people with dementia ^9^ ^,^ ^31^ ^,^ ^32^, with an effect size (ES) similar to that found by pharmacological studies (physical activity: standardized mean differences - SMD = 0.41, 95% confidence interval - 95%CI: 0.24-0.58; pharmacological studies: SMD ≤ 0.51, 95%CI: 0.35-0.67) ^9^ ^,^ ^33^. The umbrella reviews acknowledged some limitations in the current evidence restricting our knowledge of the most effective exercise characteristics to improve physical and mental symptoms in people with dementia. However, there is sufficient evidence to state that older adults with dementia benefit from physical activity ^28^.

A network meta-analysis showed that aerobic, resistance, and mind-body exercises could improve cognitive function in people with mild cognitive impairment and dementia ^26^. The authors also showed that resistance training was the most effective intervention for people with dementia compared to aerobic, multicomponent, and mind-body exercises ^26^. Moreover, another meta-analysis showed that home-based physical exercise could improve cognitive function (ES = 0.71; 95%CI: 0.43-0.99) and functional capacity (ES = 2.24; 95%CI: 1.80-2.68) and reduce neuropsychiatric symptoms (ES = 0.37; 95%CI: 0.17-0.57) and caregivers’ burden (ES = 0.63; 95%CI: 0.32-0.94) ^30^. Finally, a systematic review with meta-analysis showed that aerobic (SMD = 0.24) and resistance (SMD = 0.18) training could improve physical function in older adults in residential care ^34^. Moreover, a moderate-to-high effect was observed in studies including older adults with cognitive impairment (SMD = 0.44), with dependence in activities of daily living (SMD = 0.40), and in older adults with pre-frailty or frailty (SMD = 0.65) ^34^.

Although evidence supports the benefits of exercise for people with dementia, it is essential to acknowledge specific gaps in our understanding. For example, the significant heterogeneity observed in meta-analyses concerning exercise interventions and sample characteristics limits our knowledge regarding the most effective exercise program in intensity, frequency, and type. A recent meta-analysis suggested two to three weekly sessions of multi-component and aerobic training, each session lasting 60 minutes, to improve cognitive and physical capacity in people with dementia ^35^.

Additionally, most RCTs have been conducted in high-income countries, requiring further studies in cognitively diverse populations to better comprehend the impact of exercise on cognitive function. Given the heterogeneity of disease stages observed across meta-analyses on exercise in people with dementia, exploring the effects of exercise for each stage of the disease may enhance our understanding.

Considering the evidence, governments are expected to promote physical activity as a complementary therapy for people with dementia via public health initiatives, including a national plan to combat dementia. Instead of generic recommendations, national plans to combat dementia can address dementia-related issues, respecting each country’s culture and sociodemographic characteristics.

We reviewed national plans to combat dementia worldwide to identify the presence of initiatives to promote physical activity for people with dementia. After retrieving the national plans, we systematically searched for any mention of physical activity as a nonpharmacological and complementary therapy for people with dementia. To keep focus on national plans that promote physical activity for people with dementia, we did not include documents in which physical activity was only mentioned as a preventive strategy. For national plans published in languages other than English, Spanish, or Portuguese, online tools were used to identify the most appropriate terms for physical activity, exercise, and walking.

**Figure 1:**
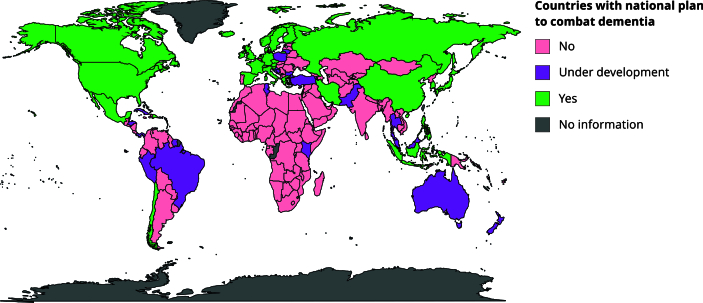
Countries with national plan to combat dementia.

Source: data was collected from the World Health Organization ^39^ and the Alzheimer’s Disease International ^40^.

From 194 countries, we identified 35 (18%) with national dementia plans: 20 (57.1%) in Europe, 10 (28.6%) in Asia, three (8.6%) in North America, and two (5.7%) in South America and the Caribbean (Figure 1). Only the following eight (22.9%) countries, all high-income countries, recommended physical activity for people with dementia: Chile, Denmark, France, Luxembourg, Norway, Singapore, Sweden, and the United States. For example, the Chilean plan reinforces the pivotal role of physical activity in preventing and managing disease ^36^. The Norwegian document ^37^ also highlights the importance of good architecture and planning to stimulate physical activity. Also, starting in January 2020, cities should offer activities for people with dementia living at home, including physical activity, as part of an inclusion strategy that contributes to mastery, meaningfulness, and good experience for these individuals ^37^. The Singaporean dementia plan promoted the Peer-to-Peer Support Group, where Zoom videoconferences with music, social engagement, health education, and exercise were offered to minimize the harmful consequences of social distancing restrictions on health ^38^.

Generally, the literature indicates that physical activity is a safe and effective complementary therapy for people living with dementia. In contrast, only 35 high-income countries designed national dementia plans that recommend physical activity for this population. This finding mirrors the small number of RCTs with physical activity in people with dementia in low- and middle-income countries and those with varying cognitive reserves. We do not intend to imply that exercise or any form of physical activity can replace traditional and innovative therapeutic methods for dementia. Instead, we emphasize the importance of promoting physical activity for this population. Older individuals with mild cognitive impairment and dementia often bear multiple comorbidities, such as cardiovascular disease and diabetes. Therefore, prescribing physical activity as a therapeutic intervention can target various therapeutic goals while minimizing side effects, and it can be tailored to individual preferences, akin to pharmacotherapy.
